# The effect of aging, hearing loss, and tinnitus on white matter in the human auditory system revealed with fixel-based analysis

**DOI:** 10.3389/fnagi.2023.1283660

**Published:** 2024-01-09

**Authors:** Veronika Svobodová, Oliver Profant, Antonín Škoch, Jaroslav Tintěra, Diana Tóthová, Martin Chovanec, Dora Čapková, Josef Syka

**Affiliations:** ^1^Department of Auditory Neuroscience, Institute of Experimental Medicine of the Czech Academy of Sciences, Prague, Czechia; ^2^Department of Otorhinolaryngology and Head and Neck Surgery, 1st Faculty of Medicine, Charles University in Prague, University Hospital Motol, Prague, Czechia; ^3^Department of Otorhinolaryngology, 3rd Faculty of Medicine, Charles University in Prague, University Hospital Královské Vinohrady, Prague, Czechia; ^4^Department of Radiodiagnostic and Interventional Radiology, Institute of Clinical and Experimental Medicine, Prague, Czechia

**Keywords:** aging, hearing loss, tinnitus, white matter, human auditory system, fixel-based analysis

## Abstract

**Introduction:**

Aging negatively influences the structure of the human brain including the white matter. The objective of our study was to identify, using fixel-based morphometry, the age induced changes in the pathways connecting several regions of the central auditory system (inferior colliculus, Heschl's gyrus, planum temporale) and the pathways connecting these structures with parts of the limbic system (anterior insula, hippocampus and amygdala). In addition, we were interested in the extent to which the integrity of these pathways is influenced by hearing loss and tinnitus.

**Methods:**

Tractographic data were acquired using a 3 T MRI in 79 volunteers. The participants were categorized into multiple groups in accordance with their age, auditory thresholds and tinnitus status. Fixel-based analysis was utilized to identify alterations in the subsequent three parameters: logarithm of fiber cross-section, fiber density, fiber density and cross-section. Two modes of analysis were used: whole brain analysis and targeted analysis using fixel mask, corresponding to the pathways connecting the aforementioned structures.

**Results:**

A significantly negative effect of aging was present for all fixel-based metrics, namely the logarithm of the fiber cross-section, (7 % fixels in whole-brain, 14% fixels in fixel mask), fiber density (5 % fixels in whole-brain, 15% fixels in fixel mask), fiber density and cross section (7 % fixels in whole-brain, 19% fixels in fixel mask). Expressed age-related losses, exceeding 30% fixels, were particularly present in pathways connecting the auditory structures with limbic structures. The effect of hearing loss and/or tinnitus did not reach significance.

**Conclusions:**

Our results show that although an age-related reduction of fibers is present in pathways connecting several auditory regions, the connections of these structures with limbic structures are even more reduced. To what extent this fact influences the symptoms of presbycusis, such as decreased speech comprehension, especially in noise conditions, remains to be elucidated.

## Highlights

- Direct connection between auditory and limbic systems.
- Pronounced impact of aging on the auditory pathway compared to the rest of the brain.
- No effect of hearing loss or tinnitus on auditory and limbic connections.

## 1 Introduction

Age-related hearing loss, presbycusis, represents a serious complication of healthy aging, and with the increasing duration of human life, the understanding of its pathogenesis becomes even more urgent. Undoubtedly, the basic pathological alterations in presbycusis are present in the inner ear, but, as recent studies show, there is a substantial central component of presbycusis present that comprises not only changes occurring in the central auditory system but also in many non-auditory structures. For example, Lin et al. (2014) observed that older adults experiencing hearing impairment exhibited a hastened reduction in the overall brain volumes and decreased regional volumes within the right temporal lobe including the superior, middle, and inferior temporal gyri as well as the parahippocampusm when compared to individuals with normal hearing. We found in elderly subjects that the gray matter volume and thickness is decreased in the Heschl's gyrus (HG), planum temporale (PT) and gyrus frontalis superior but not in the visual cortex (V1; Profant et al., 2014). Recently, we extended the morphometric study to several limbic structures [anterior insula (Ins), amygdala (Amg), and hippocampus (HP)] and included also the effect of tinnitus (Profant et al., 2020). Across all cortical structures, the thickness of gray matter diminished notably with advancing age, with no discernible impact on hemispheric laterality (the differences between the left and right hemispheres). The rate of decline in gray matter thickness was accelerated in the Heschl's gyrus (HG), planum temporale (PT), and insula compared to the parahippocampus and primary visual cortex (V1). Hearing loss only marginally resulted in a reduction of the cortical surface in the HG. Tinnitus was associated with a marginal reduction of the insular surface and led to an enlargement in the volumes of the amygdala (Amg) and hippocampus (HP), while the structure of the auditory system experienced minimal effects. Belkhiria et al. (2020) investigated alterations in brain structure linked to compromised functionality in daily activities among individuals with age-related hearing loss and discovered that atrophy in the insula (Ins), amygdala (Amg), and various temporal regions was associated with functional limitations, apathy, and language deficits in presbycusis with cochlear dysfunction.

The first study with diffusion tensor imaging in subjects with sensorineural hearing loss (Chang et al., 2004) reported reduced fractional anisotropy (FA) values within the fiber tracts traversing the superior olivary nucleus, lateral lemniscus, inferior colliculus (IC) and auditory radiation. The variability among participants in this study restricted the ability to fully intepretthe the results. Later Lin et al. (2008) described, using the same method, reduced FA values in fiber tracts connecting the lateral lemniscus and IC in subjects with hearing loss. However, reduced FA values in this region were not confirmed in the study by Husain et al. (2011) who used DTI and voxel-based-morphometry to characterize neuroanatomical changes in subjects with deteriorated hearing and tinnitus that showed decreased FA in several other brain tracts: superior and inferior longitudinal fasciculi, corticospinal tract, inferior fronto-occipital tract, superior occipital fasciculus and anterior thalamic radiation. All changes were present only in the right hemisphere. Previously, we found only limited changes (increased values of the axial vector of the diffusion and an increase of both radial vectors) within the central section of the auditory pathway above the IC associated with aging (Profant et al., 2014). Recently, Koops et al. (2021) observed an inverse correlation between age and axonal density in the bilateral acoustic radiation connecting the medial geniculate body with the primary auditory cortex.

However, in voxels with multiple and crossing fiber bundles, DTI cannot accurately allocate differences in the white matter to a specific tract, and even if changes in FA and MD occur, they cannot be bound to a specific pathology (neuronal loss, neuronal atrophy, loss of white matter, etc.). Therefore, a higher order diffusion-weighted model was recently developed that resolves the problem with multiple fiber populations within voxels. This new approach is called fixel based analysis (FBA) and is based on the fact that diffusion weighted images encapsulate data about the existence of multiple fiber bundles (fixels) within a voxel (Raffelt et al., 2017).

We have previously documented functional changes in the limbic system and auditory cortex (Fuksa et al., 2022) associated with tinnitus and presbycusis. Therefore, the primary objective of this study was to examine the relationship between aging, hearing loss, the presence or absence of tinnitus, and their impact on the white matter of connecting tracts. Our hypothesis was that the presence of tinnitus would strengthen connections between the auditory and limbic systems, aging would generally lead to white matter atrophy, and hearing loss would adversely affect the auditory tract.

For this study we employed fixel-based-analysis to identify previously mentioned alterations of the white matter in the pathways connecting IC, HG, and PT as well as the pathways connecting these auditory structures with parts of the limbic system (anterior Ins, HP, and Amg).

## 2 Methods

### 2.1 Subjects

Seventy-nine participants were included in the study, 40 males and 39 females aged between 19 and 87 years (mean age 49.9, median 54). They had either normacusis or a sensorineural type of hearing loss, clinically symmetrical hearing (asymmetry up to 20 dB between corresponding frequencies), had never experienced chronic vertigo, ear or brain surgery, nor chemotherapy. Prospective participants with a medical history of intracranial tumors, head trauma, endolymphatic hydrops, diabetes, and neurological pathology were not included in the study. The structural MRI examination was utilized to screen for any detectable neuropathology. None of the patients exhibited motor speech disorders as characterized by Utianski et al. (2019). Participants with tinnitus included in the study had suffered from tinnitus for a minimum of 6 months, stated the laterality of the tinnitus (right, left, without side determination—intracranial), the tinnitus status was evaluated by tinnitus handicap inventory (THI) questionnaire (Newman et al., 1996). All participants had no contraindication for safe MRI scanning. The study was approved by the Ethics Committee of the University Hospital Motol. All participants attended the study voluntarily after signing informed consent.

### 2.2 Assessment of auditory function

Otoscopy and tympanometry were carried out in every participant to exclude anatomical and functional pathology of the outer and the middle ear and to remove potential cerumen, followed by high-frequency pure tone audiometry comprising of all commonly examined frequencies up to 8 kHz, and also frequencies of 10, 12.5, and 16 kHz. Acoustical signals were delivered via Sennheiser HDA 200 high-frequency audiometric headphones. The audiometric apparatus was custom-made, a high-fidelity audio device (RME Fireface) in conjunction with a tailor-made programmable attenuator. This arrangement facilitated digital-to-analog conversion, attenuation/amplification of measurement signals, communication between the experimenter and the participant, and acquisition of the participant's responses through a user-friendly interface with backlit buttons (Arturia BeatStep). The system was governed by custom software developed within the Matlab environment, encompassing essential functions such as generating and playing back digital measurement signals, capturing participant responses, and conducting fundamental data analysis. The equipment underwent calibration in adherence to ISO 389-5, ISO 389-8, ISO 8253-3, and IEC 60645-3 standards, employing the Brüel and Kjær 4153 Artificial Ear. Pure tone average (PTA) of 500 Hz, 1 kHz, 2 kHz, and 4 kHz, and the average of all examined frequencies (PTA compl) were used to summarize the hearing function of every participant. Due to the inclusion criteria of symmetrical hearing, the data from both ears were combined. Audiograms were compared with average audiograms corresponding to the particular age group, as detailed in our prior research (Jilek et al., 2014). Audiograms within a range of twice the standard deviation (2 × SD) were regarded as physiological. Conversely, audiograms exceeding this 2 × SD threshold were classified as indicating hearing pathology. Participants were categorized into six groups according to their age, hearing threshold, and presence of tinnitus. Young controls with normal hearing and no tinnitus (Y-NH-NT)–10 males, 15 females, mean age 27.9 (median 26, 21–43 years old), young with normal hearing and tinnitus (Y-NH-T)–11 males, four females, mean age 32.7 (median 30, 19–50 years old), old with normal hearing and no tinnitus (O-NH-T)—two males, seven females, mean age 68.6 (median 71, 61–74 years old), old with hearing loss and no tinnitus (O-HL-NT)—nine males, three females, mean age 73.9 (median 74, 63–87 years old), old with normal hearing and tinnitus (O-NH-T)—five males, seven females, mean age 66.2 (median 67, 54–81 years old), and old with hearing loss and tinnitus (O-HL-T)—three males, three females, mean age 68.5 (median 67.5, 63–81 years old).

### 2.3 Assessment of cognitive function

All the elderly patients underwent the Montreal Cognitive Assessment (MoCA) test. Eight patients scored below 26, the threshold for mild cognitive impairment. The lowest MoCA score recorded was 22, indicating that none of the participants had clinically relevant dementia.

### 2.4 MRI data acquisition

The MRI scanning was performed at the Institute of Clinical and Experimental Medicine in Prague on a 3 T Trio Siemens scanner (Erlangen, Germany). A 12- channel head coil was used, software version syngo MR B17.

DWI data were acquired by a Spin-Echo EPI sequence with TR/TE = 8,300/106 ms, matrix 86 × 96, 2.5 × 2.5 × 2.5 mm^3^ voxel size, phase encoding direction AP, 1 volume with b-value 0, 64 directions in b = 1,100 s/mm^2^, 64 directions in b = 2,500 s/mm^2^, bandwidth 1,736 Hz/pixel in frequency encoding direction, GRAPPA acceleration factor 2 in phase encoding direction, reference lines 32, acquisition time 18:17. An additional two volumes with b = 0 in both AP and two volumes with b = 0 in PA phase encoding direction with otherwise identical parameters were acquired.

For an estimation of the intracranial volume, needed as a covariate in statistical models, a 3D structural image was obtained utilizing the magnetization prepared rapid acquisition gradient echo (MPRAGE) sequence with the parameters (TI – inversion time) TI/TR/TE = 900/2,100/2.63 ms, flip angle 10°, 1 average, phase partial Fourier 6/8, matrix 256 × 256 × 256, voxel size 0.86 × 0.86 × 0.86 mm^3^, phase oversampling 40%, without slice oversampling, bandwidth 290 Hz/pixel, echo spacing 6.3 ms, without parallel imaging, prescan normalize, eliptical filter, acquisition time 9:26.

Structural imaging by 2D FLAIR and 3D T1 MPRAGE and 3D T2 SPACE were performed, and images were evaluated to rule out other structural abnormalities.

### 2.5 DWI preprocessing

The data underwent processing by software suite Mrtrix, version 3.0.1 (Tournier et al., 2019), and FSL tools, version 6.0.3 (Jenkinson et al., 2012). The preprocessing started by denoising using random matrix theory (Veraart et al., 2016) using an enhanced noise estimator implemented by Cordero-Grande et al. (2019). Gibbs-ringing artifacts were eliminated employing the technique outlined by Kellner et al. (2016). Susceptibility-induced image distortion was estimated by FSL TOPUP (Andersson et al., 2003; Jenkinson et al., 2012). Correction for movement (including slice-to-volume motion correction), eddy-current-induced distortions, outlier replacement and susceptibility-induced distortions were done by the FSL EDDY tool (Andersson and Sotiropoulos, 2016; Andersson et al., 2016, 2017).

### 2.6 Fixel-based analysis

The fixel-based analysis pipeline steps were carried out according to the Mrtrix documentation (https://mrtrix.readthedocs.io; Raffelt et al., 2017). Briefly, the steps involved bias field correction, average tissue response function estimation (Dhollander et al., 2016), image upsampling, brain mask estimation, fiber orientation distribution estimation (by using multi-shell multi-tissue spherical deconvolution; Tournier et al., 2007; Jeurissen et al., 2014), joint bias field correction and intensity normalization, generation of study-specific unbiased FOD template, registering all subject FOD images to the FOD template, computation of the template mask, computation of the white matter template analysis fixel mask, warping FOD images to the template space, segmentation of FOD images and estimation of their apparent fiber density (FD), fixel reorientation, assignment of subject fixels to template fixels, computation of fiber cross-section metric (FC), computation of a combined fiber density and cross-section measure (FDC), whole-brain fiber tractography on FOD template with subsequent reduction of tractography biases by SIFT method (Smith et al., 2012), fixel-fixel connectivity matrix determination, and smoothing of fixel data using fixel connectivity.

For statistical analysis, two modes of fixel set were used:

(1) “Whole-brain” where the original fixel set from the whole brain derived by standard fixel-based analysis method was used.
(2) Restricted mask of fixels involved by white matter connections hypothetically involved in hearing and tinnitus. To get this mask, the tractography between the specified brain areas was performed and the fixels relevant to the streamlines were identified.

Namely, “restricted” fixel mask was derived by the following method.

**Figure 1:**
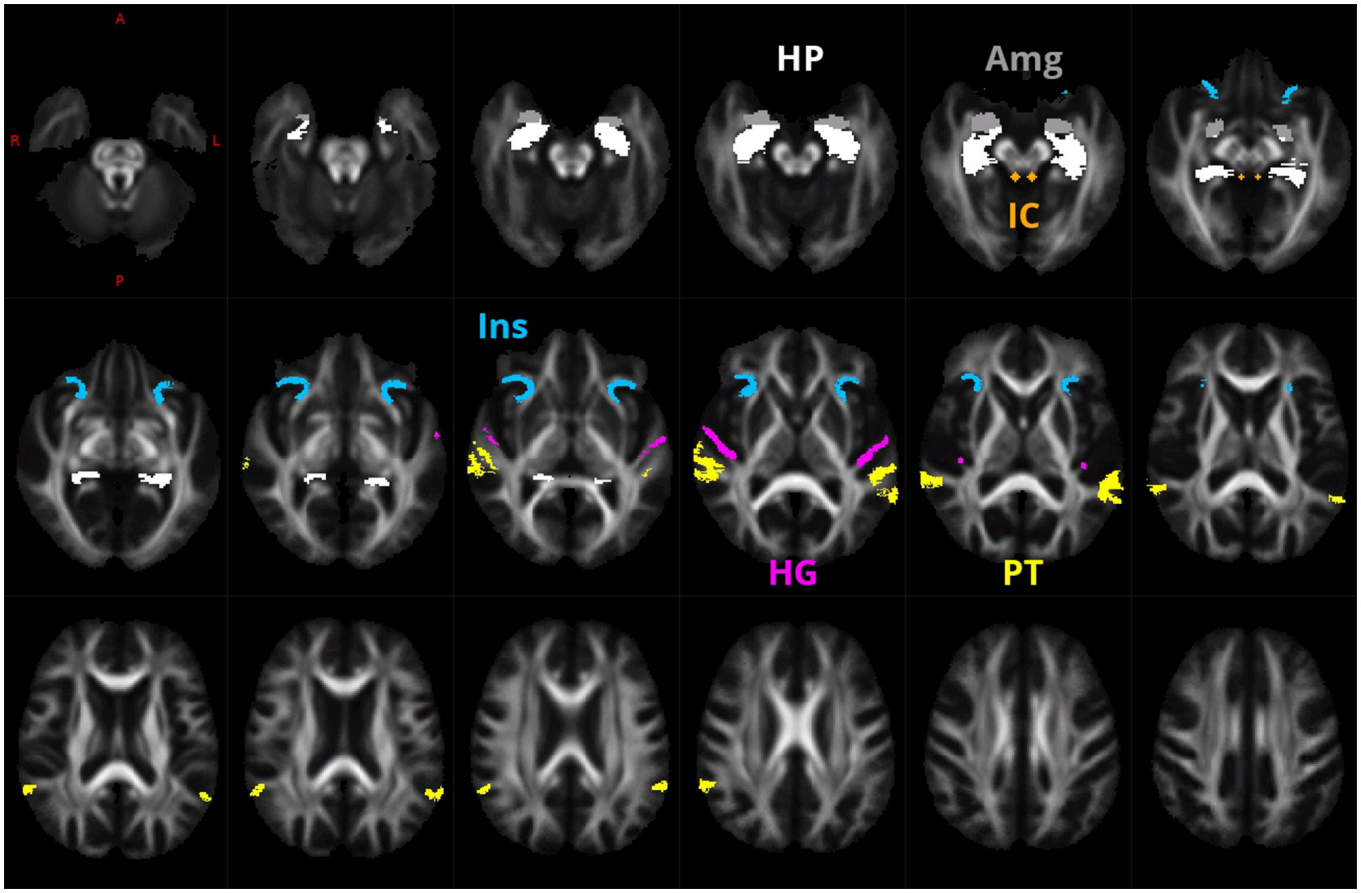
ROIs of HG (purple), PT (yellow), anterior Ins (blue), HP (white), Amg (gray), and IC (green) that were used for the creation of the fixel mask.

ROIs of Heschl gyrus (HG), planum temporale (PT), anterior insula (Ins), hippocampus (HP), amygdala (Amg), and inferior colliculus (IC; Figure 1) were determined by the processing of Collin27 single subject template (Holmes et al., 1998) by FreeSurfer version 7.1.1 (Fischl, 2012) using Destrieux atlas (Fischl et al., 2004) and hippocampal subfields/nuclei of amygdala FreeSurfer modules (Iglesias et al., 2015; Saygin et al., 2017). The FA images were estimated from each subject. They were registered together by using warps estimated in FOD template generation, and voxel-wise FA median over all subjects was computed to create a study-specific FA template, residing in the same space as the FOD template. This then created the FA template affinely registered to the FMRIB58 FA template (which resides in the MNI space as well as the Colin27 template). This transform was used to bring a mask of selected ROIs from Collin27/MNI space to our study-specific FOD template space. Additionally, a mask of inferior colliculus was manually defined on the FA template as a sphere of 3 voxels in diameter.

Then, a probabilistic tractography on the FOD template, between the selected 26 pairs of ROIs (as defined in Supplementary Figures 1–5, totally 26 ROI pairs) was performed.

The 26 tractograms were added together and for each template fixel the number of traversing streamlines of this summed tractogram was determined. Then, for the fixel mask we selected only those fixels who had, from the summed tractogram, more than 26 streamlines assigned.

We utilized a general linear model framework to establish fixel-wise relationships between fixel parameters (FD, FC, and FDC) and explanatory variables. The model's matrix of explanatory variables comprised an intercept, categorical variables for hearing and tinnitus, and continuous covariates like age and total intracranial volume.

We tested both-sided hypotheses for model coefficients related to hearing, tinnitus, and age. The hearing hypothesis examines the significance of differences between NH and HL groups, irrespective of age and tinnitus. The tinnitus hypothesis focuses on the significance of differences between NT and T groups, regardless of age and hearing. The age hypothesis assesses the significance of a non-zero slope of the linear curve fitted to the relationship between a particular fixel parameter and age, independent of tinnitus and hearing.

Total intracranial volume, determined by the FreeSurfer Samseg module (Puonti et al., 2016), was considered a covariate of no interest and not subjected to testing within the model.

Statistical maps related to specific model hypotheses underwent filtering by connectivity-based fixel enhancement (Raffelt et al., 2015). This approach, tailored to the structure of fixel maps and akin to TFCE (Smith and Nichols, 2009), incorporates the spatial relationships inherent in statistical maps, thereby enhancing result sensitivity.

Statistical significance values for each fixel and hypothesis were determined using non-parametric permutation testing of filtered statistical maps (Winkler et al., 2014). This method involved comparing test statistics with a null distribution, generated by repeatedly evaluating the linear model while randomly permuting data labels. Details of the filtering and permutation method, executed through the Mrtrix command fixelcfestats, are elaborated in Raffelt et al. (2015). Our reported results are FWE corrected and thresholded at a *p*-value of 0.05.

For improved interpretation of FBA results, we computed the spatial involvement degree of each tractogram in statistical significance. This analysis involved determining the ratio of significant fixels to the total fixels assigned to a specific tractogram.

## 3 Results

### 3.1 State of hearing

**Figure 2:**
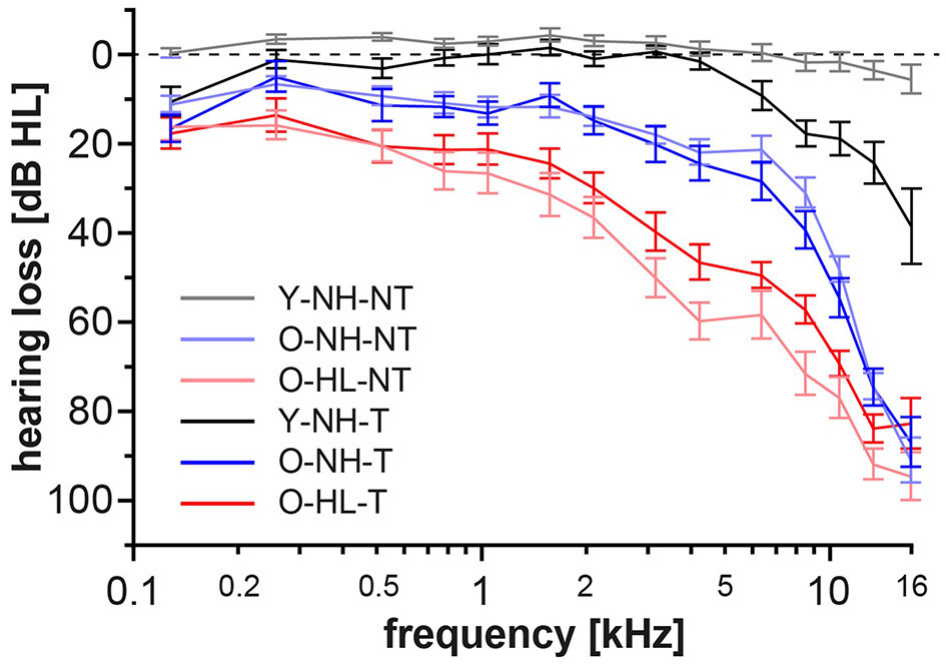
Average auditory thresholds (+/– SD) of different groups. The presence of tinnitus does not cause a significant difference in auditory thresholds of age balanced groups (*t*-test, *p* = 0.05).

In accordance of the outcomes from the high-frequency pure tone audiometry, we excluded hearing pathology in participants with normacusis and identified subgroups with hearing loss. There were four subgroups with normal hearing—Y-NH-NT, Y-NH-T, O-NH-NT, O-NH- T, hearing pathology was present in two subgroups O-HL-NT and O-HL-T (Figure 2). The average PTA and PTAcomp of all subgroups are shown in Table 1. No notable distinctions in hearing thresholds were observed when comparing the tinnitus and non-tinnitus groups, ensuring age balance (unpaired *t*-test, GraphPad Prism).

**Table 1:** Average hearing function of the participants presented as pure tone average (PTA for 500 Hz, 1 kHz, 2 kHz, and 4 kHz) and PTAcompl (for all examined frequencies).

	**PTA (dB)**	**PTAcompl (dB)**
Y-NH-NT (*n* = 25)	−1.0	1.9
Y-NH-T (*n* = 15)	4.4	9.7
O-NH-NT (*n* = 12)	15.3	27.2
O-NH-T (*n* = 9)	15.9	25.9
O-HL-NT (*n* = 12)	35.4	47.1
O-HL-T (*n* = 6)	40.0	45.7

The tinnitus groups comprised 30 subjects, the average duration of the tinnitus was 4.3 years (from 0.5 to 8 years). Table 2 shows the distribution of the laterality of the tinnitus and the outcome of the THI questionnaire of the tinnitus in this group. In addition, Table 2 shows an overview of the laterality, duration, and THI score in tinnitus subgroups based on the age distribution and presence of hearing loss.

**Table 2:** Distribution, duration, laterality, and severity (according to THI) of tinnitus in all tinnitus subgroups.

**Tinnitus group**	**Mean duration (yrs)**						
*n* = 30	4.3		Bilateral	Right	Left		
		Laterality	*n* = 18	*n* = 7	*n* = 8		
			Slight	Mild	Moderate	Severe	Catastrophic
		THI	*n* = 18	*n* = 10	*n* = 2	*n* = 2	*n* = 0
**Tinnitus subgroups**	**Mean duration (yrs)**						
Y-NH-T	3.8		Bilateral	Right	Left		
*n* = 15		Laterality	*n* = 6	*n* = 4	*n* = 5		
			Slight	Mild	Moderate	Severe	Catastrophic
		THI	*n* = 8	*n* = 5	*n* = 1	*n* = 1	*n* = 0
O-NH-T	3.7		Bilateral	Right	Left		
n = 9		Laterality	n = 8	n = 1	n = 3		
			Slight	Mild	Moderate	Severe	Catastrophic
		THI	n = 8	n = 4	n = 0	n = 0	n = 0
O-HL-T	5.4		Bilateral	Right	Left		
*n* = 6		Laterality	*n* = 4	*n* = 2	*n* = 0		
			Slight	Mild	Moderate	Severe	Catastrophic
		THI	*n* = 3	*n* = 2	*n* = 0	*n* = 0	*n* = 0

### 3.2 Whole-brain fixel-based analysis

**Figure 3:**
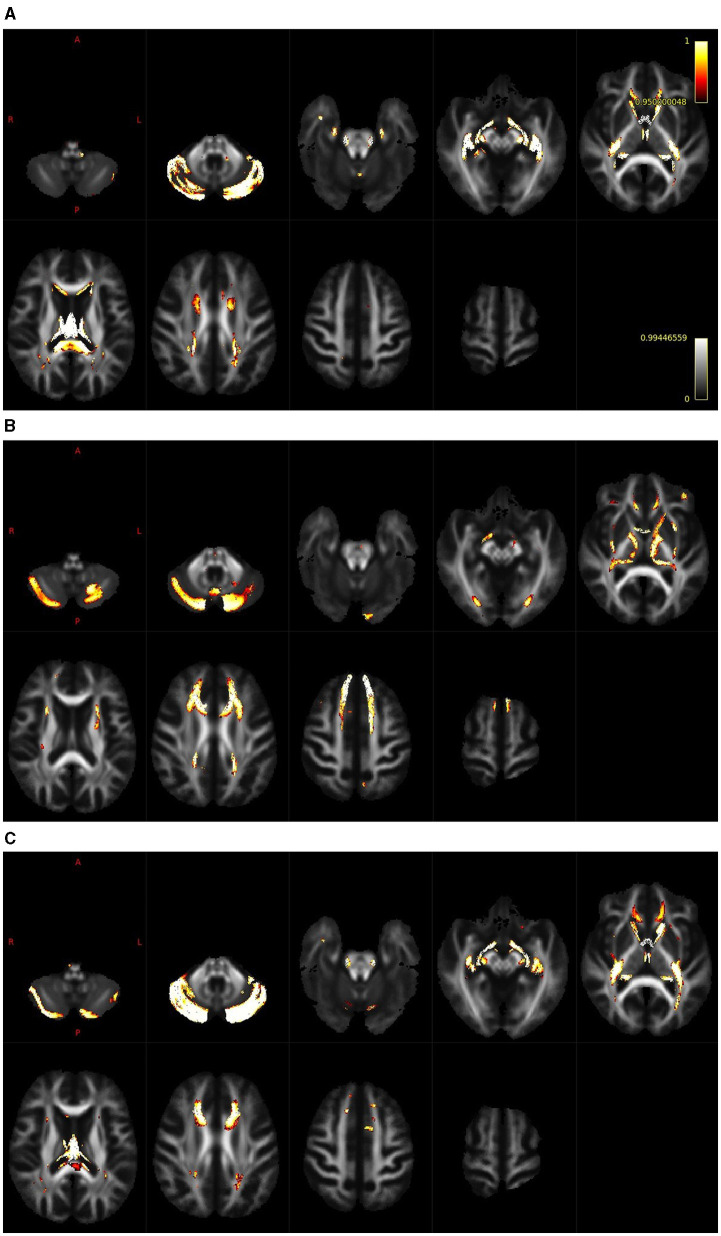
Whole brain analysis shows regions with a reduction of FD [**(A)**; cerebellum, splenium of the corpus callosum and fornix], log FC [**(B)**; cerebellum, frontal part of the corpus callosum, genu corporis callosi, internal capsula, commisura anterior and acoustic radiation] and FDC [**(C)**; cerebellum, cropus callosum and internal capsula].

The results of the whole-brain fixel-based analysis are shown in Figures 3A–C. These figures show the streamline segments associated with the fixels that expressed a significant age-related decrease for FD, log FC and FDC. Streamlines are colored by the magnitude of reductions present in aged subjects in comparison with young subjects. For the whole brain analysis 418,551 fixels were identified, an age-related reduction of FD was present in 5% of fixels, in the case of log FC the decrease was observed in 7% of fixels and in the case of FDC in 7% of fixels. Figure 3A illustrates the decreases in the FD that were present, particularly in the cerebellum, splenium of the corpus callosum and fornix. In the case of log FC (Figure 3B) major reductions occurred in the cerebellum, in the frontal part of the corpus callosum, in genu corporis callosi and in the internal capsula, commisura anterior, and acoustic radiation. Reductions in the case of FDC were less expressed and occurred in some parts of the structures where reductions were present in the log FC and FD (Figure 3C).

### 3.3 Tract of interest analysis

Fixel based analysis was performed in tracts connecting six ROIs selected on the conclusions drawn from our previous studies (Profant et al., 2014, 2020). Six ROIs comprised the following structures: IC, HG, PT, HP, Amg, and anterior Ins (see Figure 1). Individual pathways are shown in Supplementary Figures 1–5. The results of the probabilistic tractography on the FOD template between the selected 26 pairs of ROIs (as shown in Supplementary Figures 1–5) show that the effects of hearing and/or tinnitus did not reach significance, and significant changes were connected with aging only.

**Figure 4:**
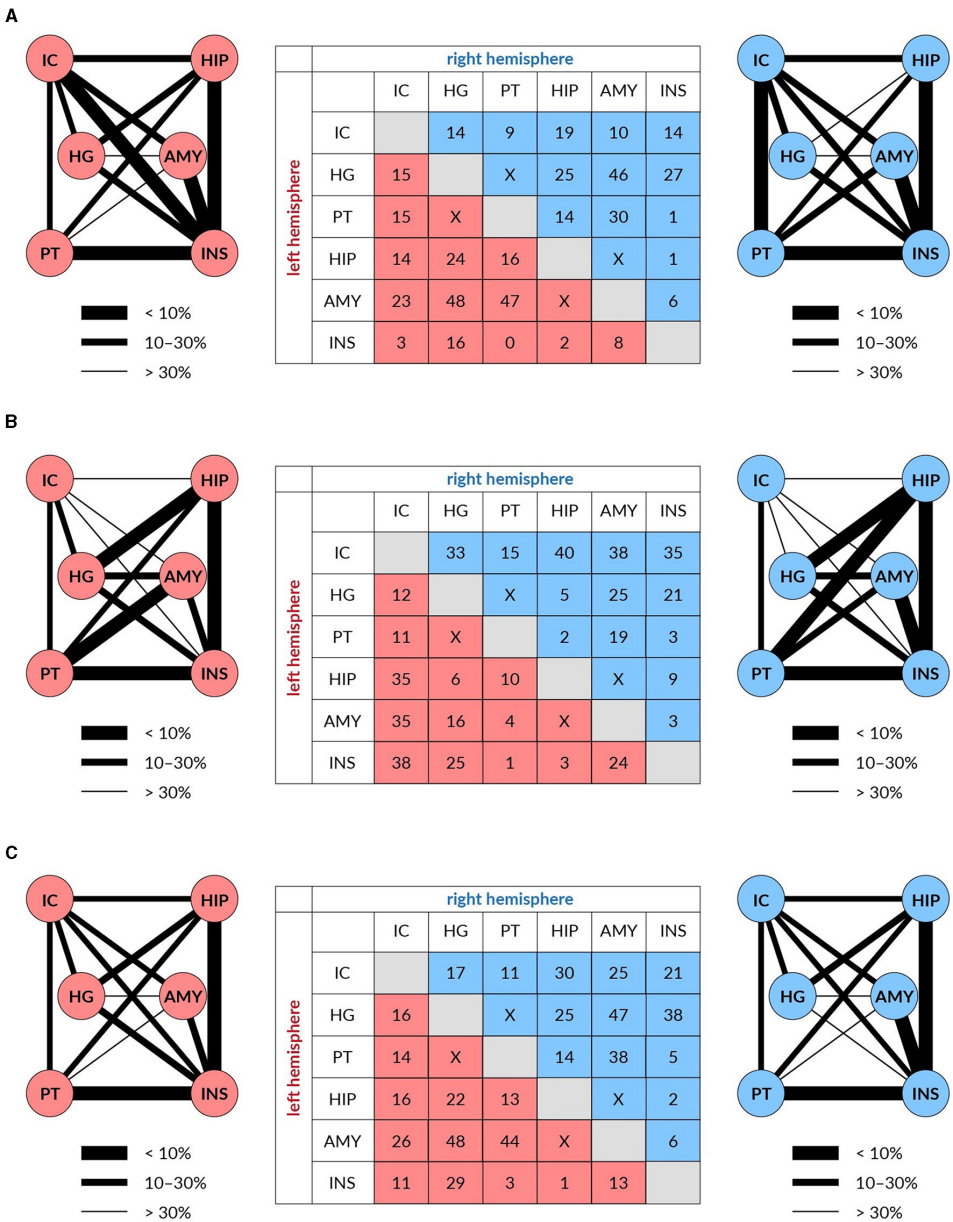
The reduction of white matter tracts connecting auditory (IC, HG, and PT) and limbic structures (HP, Amy, and Ins) in both hemispheres (left in red and right in blue) expressed in % (table) and as the thickness of the connecting lines FD **(A)**, log FC **(B)**, and FDC **(C)**.

For fiber density, the age-related reduction of fixels for all used masks together amounted to 15% of fixels. This reduction was, however, unevenly distributed, as is shown in Figure 4A. The most expressed reductions of fixels were not present in the pathways linking components of the central auditory system, particularly those connecting auditory structures with non-auditory ones. For example, the largest reductions were found in the pathway connecting HG with Amg (both in the left and right hemispheres) and in the pathway connecting PT with Amg on the left side and HG with Amg on the right side.

For fiber cross-section, the age-related reduction of fixels for all used masks amounted to 14% of fixels. The reduction was in this case also unevenly distributed, as shown in Figure 4B. The maximal decrease of fixels occurred in the pathways connecting the IC with non-auditory structures HP, Amg and anterior Ins, with the decreases being almost identical in the left and right hemispheres.

Age-related decreases of fixels in the fiber density and cross-section for all used masks appeared in 19% of fixels. The decreases were more evenly distributed however, similarly as in the case of FD and log FC, the largest decreases were present between the auditory structures as HG or PT with Amg on both hemispheres (Figure 4C).

## 4 Discussion

Our data clearly demonstrate that aging is linked to a decrease in density and a cross- section of fibers within the pathways connecting components of the central auditory system, IC, HG, and PT, as well as in the pathways connecting these auditory structures with some parts of the limbic system, i.e., Amg, HP, and anterior Ins. Fixel based analysis showed that age-related reduction concerns 5–7% of fixels in the case of whole brain analysis and between 14 and 19% of fixels when using fixel masks covering specific central auditory pathways and pathways connecting central auditory structures with parts of the limbic system. We observed no notable alterations in the white matter of the pathways examined by FBA that could be linked to hearing loss and tinnitus.

These are, according to our knowledge, the first data based on the FBA analysis that describe the complex of the central auditory pathways and their connections with the limbic system. Recently, FBA was used by Koops et al. (2021) to study the effects of hearing loss and tinnitus on the structure of acoustic radiation. They observed tinnitus-related atrophy of the left acoustic radiation near the medial geniculate body. In our study we did not find such a change in the pathway connecting the IC with the HG that accommodates connection of the inferior colliculus with the medial geniculate body and acoustic radiation. However, in agreement with our data Koops et al. (2021) described a decrease in the density of fibers in the acoustic radiation bilaterally that corresponds with our findings. In our case, the age-related loss of density of fibers in the pathway connecting IC with HG amounts to 15% on the left side and 14% on the right side.

Fixel-based analysis has recently been used in several studies (Choy et al., 2020; Zivari Adab et al., 2020; Kelley et al., 2021) to reveal age-related differences in the brain white matter in a more precise way than the previous studies performed with DTI. The decreases in the brain white matter induced by aging as described in these studies in principle correspond with our data obtained in the whole brain analysis. For instance, Kelley et al. (2021) discovered pronounced age-related variations in fiber density in certain areas like the fornix, bilateral anterior internal capsule, forceps minor, body of the corpus callosum, and corticospinal tract. Conversely, age-related differences in fiber cross-section were more noticeable in the cingulum bundle and forceps minor. In a separate study, Choy et al. (2020) observed that specific fiber bundles such as the anterior thalamic radiation, corpus callosum, and superior longitudinal fasciculus exhibited a substantial negative correlation with age only in terms of fiber density (FD) values, not in fiber cross-section (FC). On the contrary, segments of the cerebello-thalamo-cortical pathway showed a significant negative correlation with age in FC but not in FD. In our case, FBA of the whole brain revealed similar decreases in the white matter as the aforementioned studies. Similarly, like Choy et al. (2020), we observed that reductions are not necessarily identical between FC and FD. For example, decreases occurred in the cerebellum both in FC and FD, however in the corona radiata in the frontal part of the brain only significant reduction of FC was found.

Presbycusis has been, in recent years, the subject of the study of several morphometrically oriented investigations of the brain. Specifically, we examined alterations in cortical gray matter parameters using MRI. In our previous research, focusing on elderly subjects, we noted a reduction in gray matter volume and thickness in the Heschl's gyrus (HG) and planum temporale (PT; Profant et al., 2014), along with various limbic structures like the anterior insula (Ins), amygdala (Amg), and hippocampus (HP; Profant et al., 2020). Within all these structures, gray matter thickness exhibited a significant decline with age, without affecting hemispheric asymmetry. Additionally, Belkhiria et al. (2020) observed atrophy of the insula (Ins), amygdala (Amg), and other temporal regions in subjects with presbycusis, which correlated with functional decline, apathy, and language deficits. Furthermore, Ren et al. (2018) documented age-related gray matter atrophy in several auditory cortical regions, bilateral precuneus, inferior parietal lobule, the right posterior cingulate cortex, and the right insula. All these studies show, that in the aforementioned limbic structures, expressed loss of neurons occurs with aging. Such loss may also significantly contribute to decreases in the pathways connecting limbic and central auditory structures, such as IC, primary AC and PT. It remains to be elucidated, to what extent these losses in pathways connecting the aforementioned structures of the limbic system may influence the functional deterioration of hearing typical for presbycusis.

In addition to examining alterations in gray matter, age-related changes in brain white matter structure were recently investigated by DTI (Chang et al., 2004; Lin et al., 2008; Husain et al., 2011). The changes in the white matter were, however, more frequently the subject of investigation in studies concerning tinnitus (Crippa et al., 2010; Aldhafeeri et al., 2012; Benson et al., 2014). For instance, Yoo et al. (2016) revealed that white matter integrity in individuals with chronic tinnitus was directly impacted by age and was also influenced by hearing loss. Chen et al. (2020) reported slightly different results with hearing loss, having a non -significant effect on the white matter integrity and tinnitus affecting the integrity irrespective of its duration or THI burden. Our results show that the most important effect on white matter integrity is age, whereas tinnitus and hearing loss have non-significant effects. The difference between our data and the results of Yoo et al. (2016) and Chen et al. (2020) might be explained not only by the difference in the age of the volunteers, but mostly by the different method of white matter analysis. FBA presents two primary benefits when compared to other diffusion MRI analysis approaches: sensitivity to microstructure-specific properties regardless of local fiber geometry and precision in the analysis and outcomes concerning individual fiber-specific impacts (Dhollander et al., 2021, p. 1).

From a functional standpoint, all three parameters; aging, hearing loss and tinnitus induce changes in the AC, and to a smaller degree also in the parts of the limbic system (Ins and HP; Fuksa et al., 2022). The functional engagement of limbic structures within the tinnitus network is further affirmed by the discoveries of De Ridder et al. (2011) and Maudoux et al. (2012). Although we identified anatomic tracts directly connecting the auditory and limbic system, we were unable to confirm previously identified functional changes by the structural changes in tinnitus patients.

## 5 Limitations and future directions

The primary limitation of our study pertains to the number of participants and its utilization for statistical analysis. Although 79 participants might seem sufficient, dividing them into subgroups diminishes the likelihood of identifying statistically significant results. However, none of the effects are independent and cannot be treated as continuous (with exception of aging). Consequently, we maintain confidence in our chosen statistical approach.

Our current and previous studies strongly indicate that comprehending complex pathologies such as presbycusis and tinnitus necessitates analyzing not only the auditory system but also understanding the involvement of various brain regions, including those highlighted in our study: the amygdala, hippocampus, and insula. Several reports (Yeend et al., 2019; Belkhiria et al., 2020) demonstrate the connection between presbycusis and impairment of regions beyond the auditory system. Moreover, additional auditory symptoms beyond elevated auditory thresholds contribute to the main sign of central presbycusis which is deteriorated speech perception especially in noisy environment. A similar relationship, to a limited extent, exists for tinnitus. Thus, we propose that future investigations should focus on a more comprehensive understanding and characterization of both pathologies. Enhancements in MRI examinations could significantly aid in identifying potential changes.

## 6 Conclusions

Our findings demonstrated evident anatomical links between the auditory and limbic systems, showcasing an impact of aging that affects both systems to varying extents. Regrettably, we couldn't demonstrate the impacts of hearing loss or tinnitus on these systems, as neither effect was apparent throughout the brain. Nonetheless, the existence of anatomical connections between the auditory and limbic systems suggests the potential involvement of the limbic system in individuals with tinnitus.

Recent findings complement our previous reports about the functional (Fuksa et al., 2022) and gray matter (Profant et al., 2020) changes within the auditory and limbic systems induced by aging, hearing loss and tinnitus. Our study confirmed higher specificity of the FBA over DTI as regards to white matter changes within the auditory pathway above the IC, in comparison with our older report (Profant et al., 2014).

## Data availability statement

The raw data supporting the conclusions of this article will be made available by the authors, without undue reservation.

## Ethics statement

The studies involving humans were approved by Ethics Committee of the University Hospital Motol. The studies were conducted in accordance with the local legislation and institutional requirements. The participants provided their written informed consent to participate in this study.

## Author contributions

VS: Formal analysis, Investigation, Writing—original draft. OP: Writing—original draft, Writing—review & editing, Funding acquisition, Project administration, Resources. AŠ: Formal analysis, Investigation, Methodology, Writing—original draft. JT: Conceptualization, Investigation, Methodology, Supervision, Writing—review & editing. DT: Investigation, Project administration, Writing—original draft. MC: Funding acquisition, Project administration, Resources, Writing—review & editing. DČ: Investigation, Writing—original draft. JS: Funding acquisition, Supervision, Writing—review & editing.
