# Supplementary figures and images for: The effect of aging, hearing loss, and tinnitus on white matter in the human auditory system revealed with fixel-based analysis
Source: Front Aging Neurosci. 2024 Jan 9;15:1283660. doi: 10.3389/fnagi.2023.1283660 (PMC10803717; doi:10.3389/fnagi.2023.1283660)

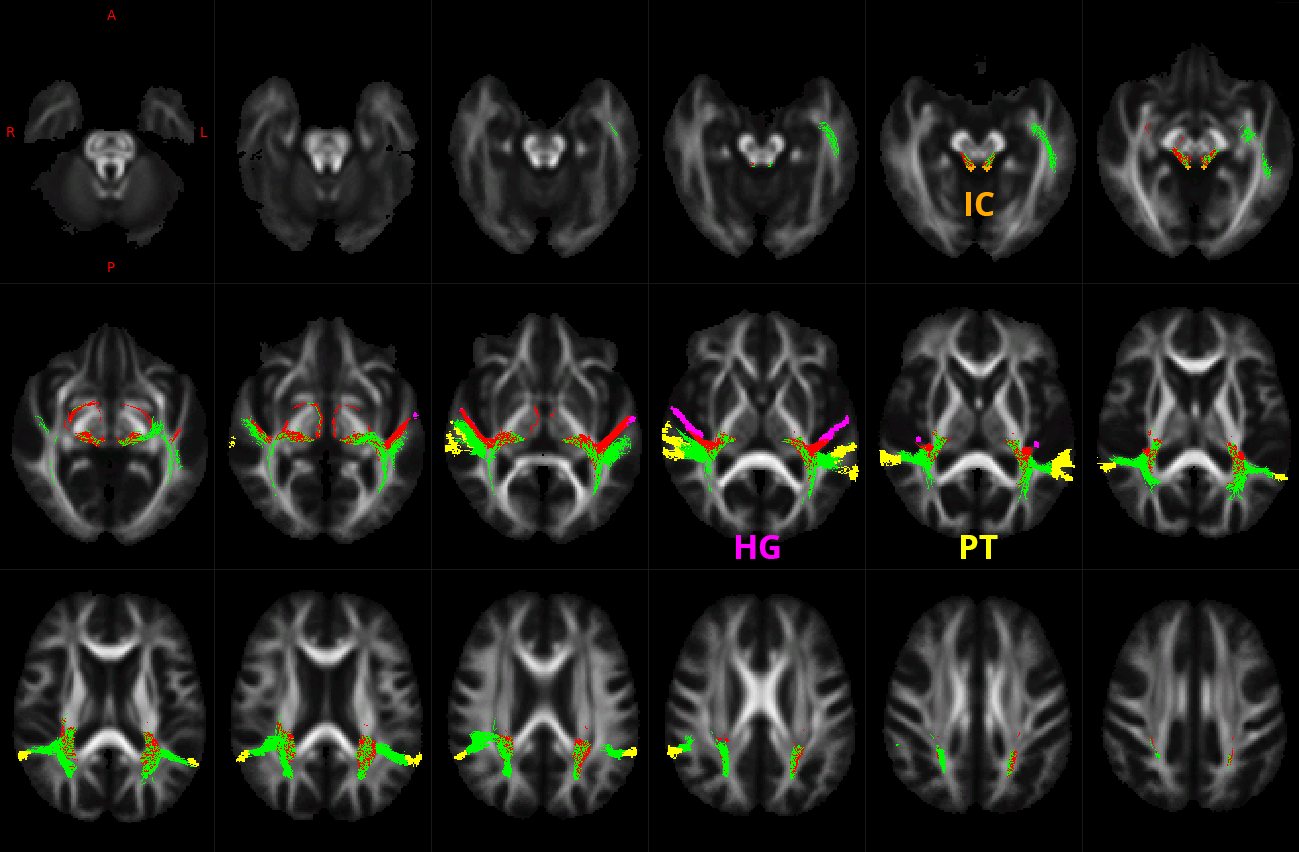

Supplement: Supplementary file 2 [file Image_1.png]

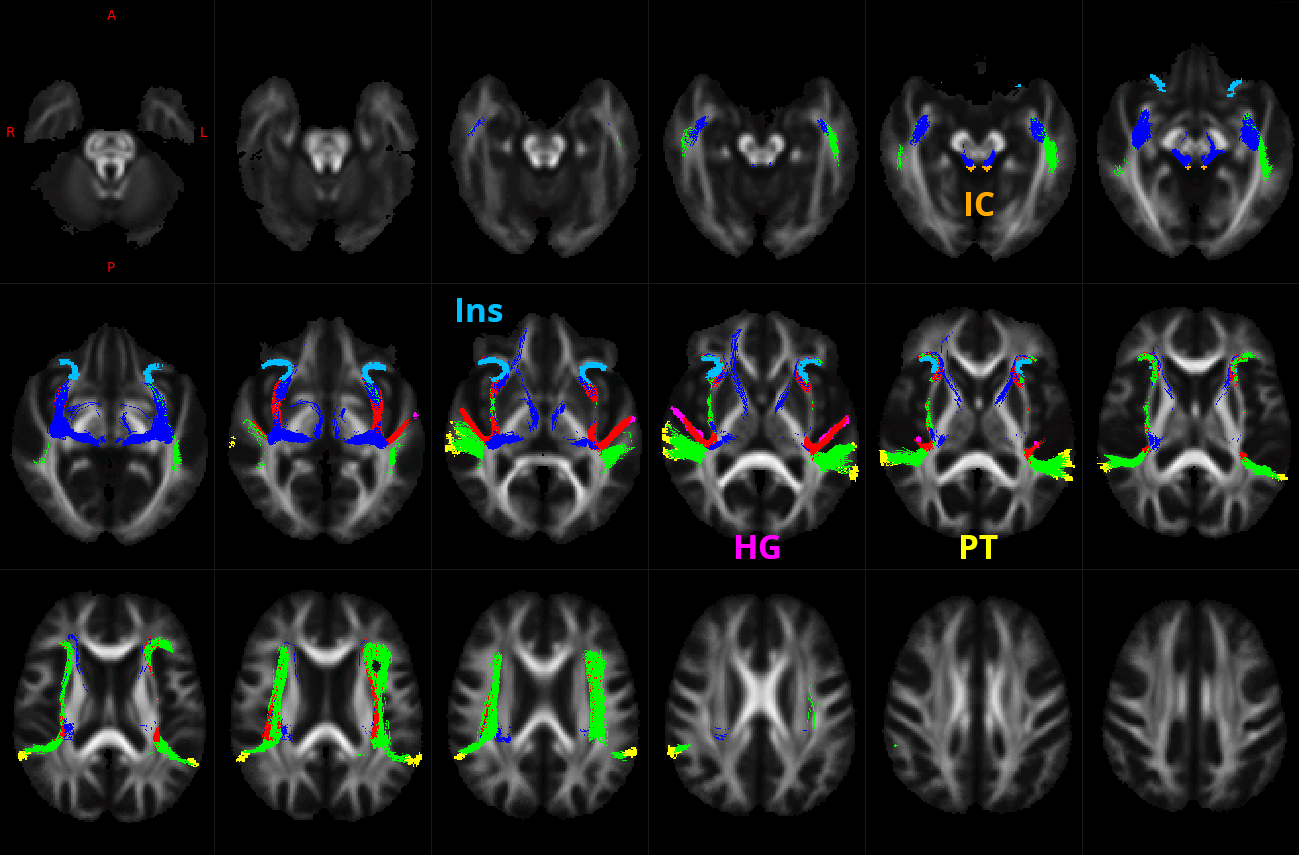

Supplement: Supplementary file 3 [file Image_2.png]

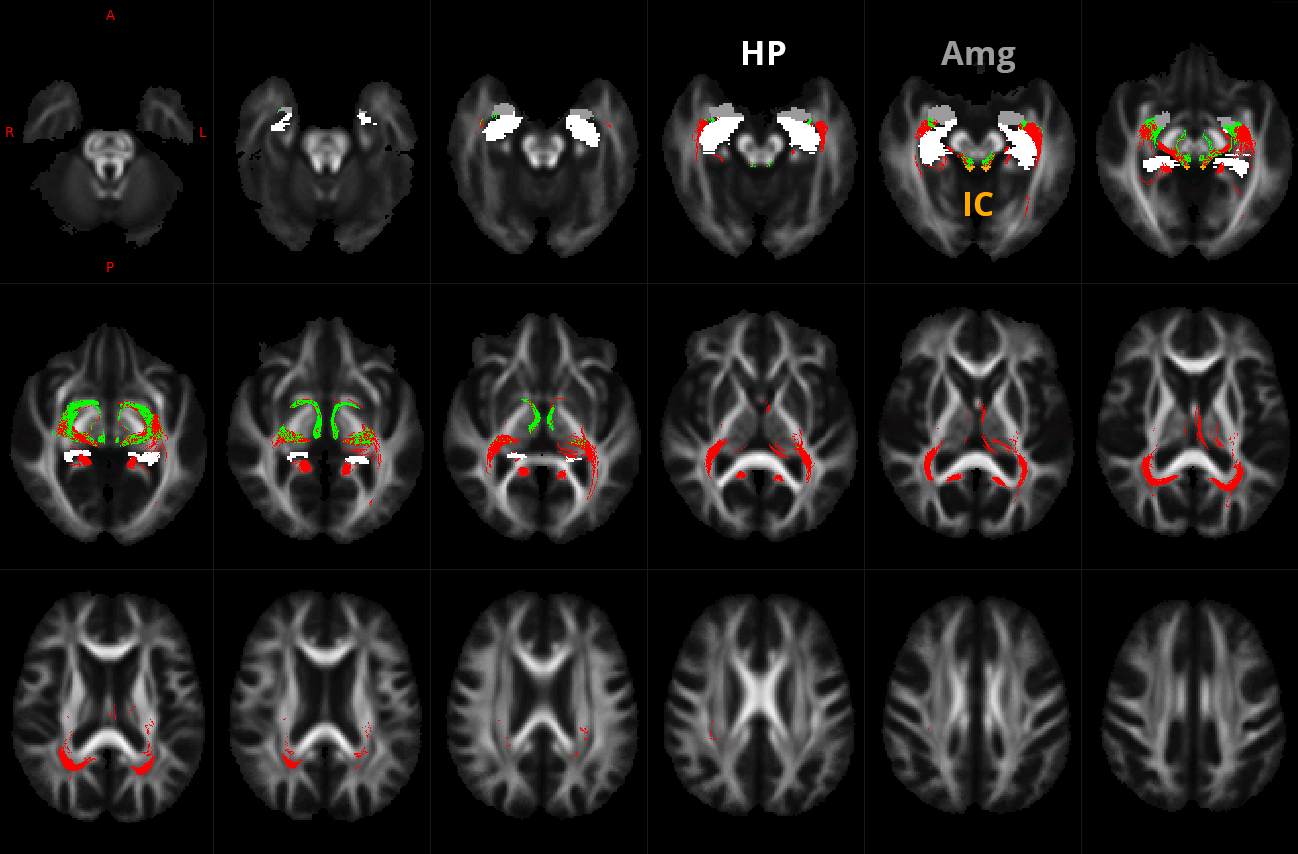

Supplement: Supplementary file 4 [file Image_3.png]

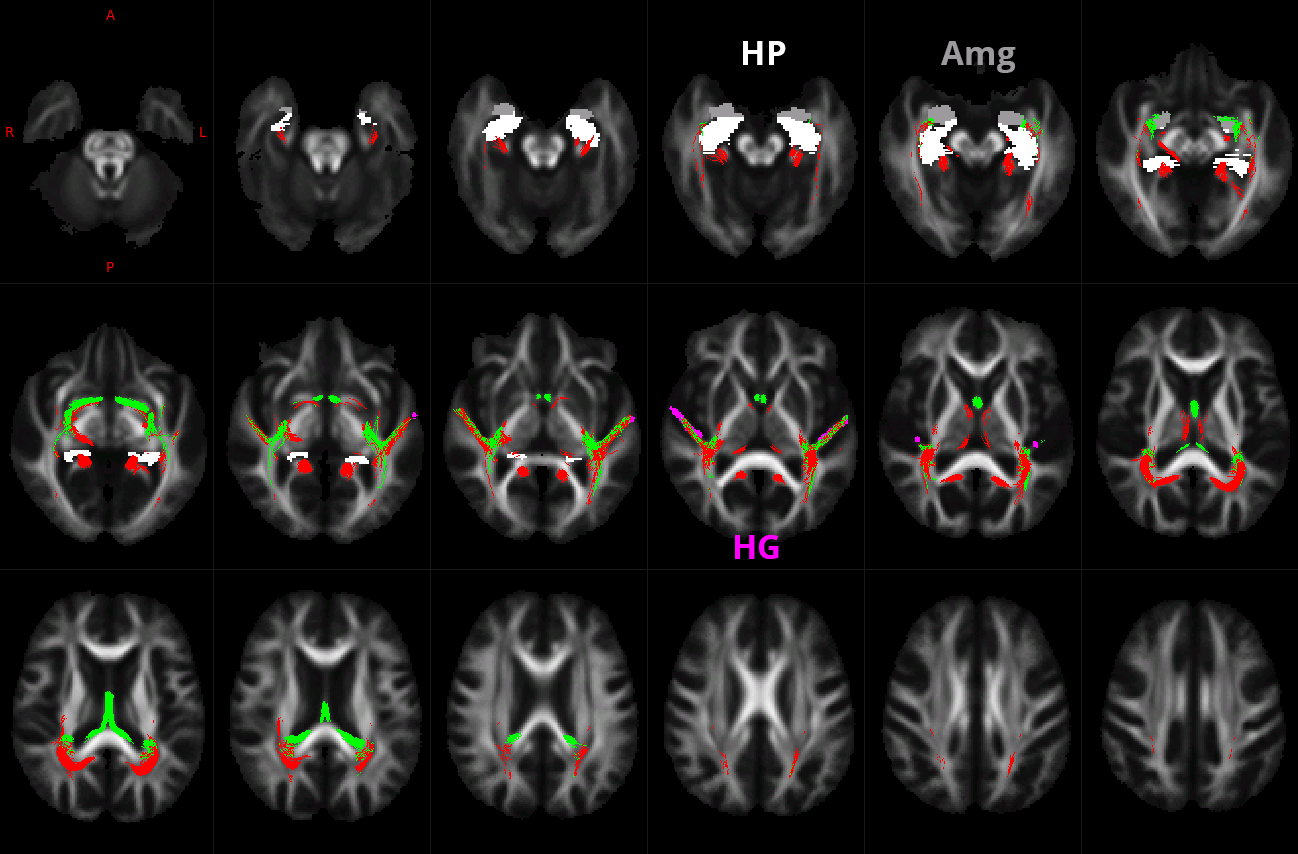

Supplement: Supplementary file 5 [file Image_4.png]

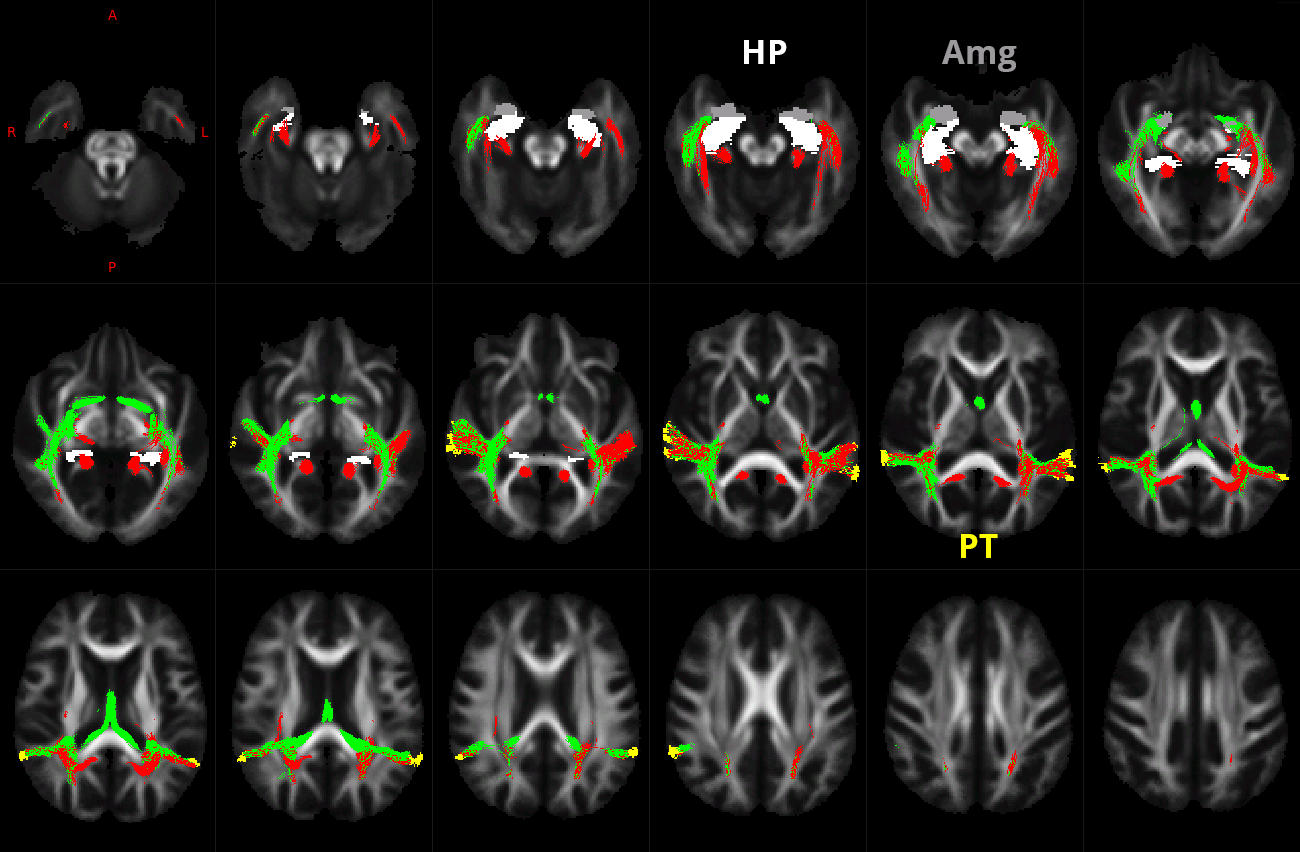

Supplement: Supplementary file 6 [file Image_5.png]
